# New online beam intensity synchronous monitoring system in scanning transmission X-ray microscopy

**DOI:** 10.1107/S1600577524012141

**Published:** 2025-02-04

**Authors:** Yuchen Jiao, Xiangzhi Zhang, Zijian Xu, Zhen Yao, Tianxiao Sun, Yufei Zhang, Bo Zhao, Zhi Guo, Yong Wang, Xiangjun Zhen, Haigang Liu, Shasha Liang, Haitao Li, Xuanyu Zhao, Jian He, Renzhong Tai

**Affiliations:** a Shanghai Institute of Applied Physics Chinese Academy of Sciences Shanghai201800 People’s Republic of China; b University of Chinese Academy of Sciences Shanghai100049 People’s Republic of China; c Shanghai Synchrotron Radiation Facility, Shanghai Advanced Research Institute Chinese Academy of Sciences Shanghai201204 People’s Republic of China; d Shanghaitech University Shanghai201210 People’s Republic of China; e The University of Texas at Austin Austin TX78712 USA; Bhabha Atomic Research Centre, India

**Keywords:** STXM, high-speed beam intensity monitoring, top-up mode, noise removal

## Abstract

The scanning transmission X-ray microscopy (STXM) platform based on synchrotron radiation has achieved nanoscale imaging with chemical sensitivity using spectro-microscopy techniques. However, the quality of STXM imaging is affected by the stability of the beam intensity. The top-up operation mode of synchrotrons to maintain a constant electron beam intensity introduces periodic fluctuations in the X-ray beam intensity, leading to notable imaging noise that decreases both contrast and precision. To address this issue, a high-speed real-time beam intensity monitoring system was designed and implemented at the BL08U1A beamline of the Shanghai Synchrotron Radiation Facility. This system utilizes an yttrium–aluminium–garnet crystal along with dual detectors having an acquisition frequency of up to 1 MHz and a synchronization error of less than 20 ns between them. This system can precisely and synchronously monitor the X-ray beam intensity variations which are used to remove noise due to electron injection from STXM images, thereby markedly improving the quality of STXM imaging.

## Abstract 2

A new online beam intensity synchronous monitoring system has been designed which can precisely and synchronously monitor X-ray beam intensity variations. This can be used to remove noise due to electron injection from STXM images, thereby markedly improving the quality of STXM imaging.

## 1. Introduction

**Figure 1:**
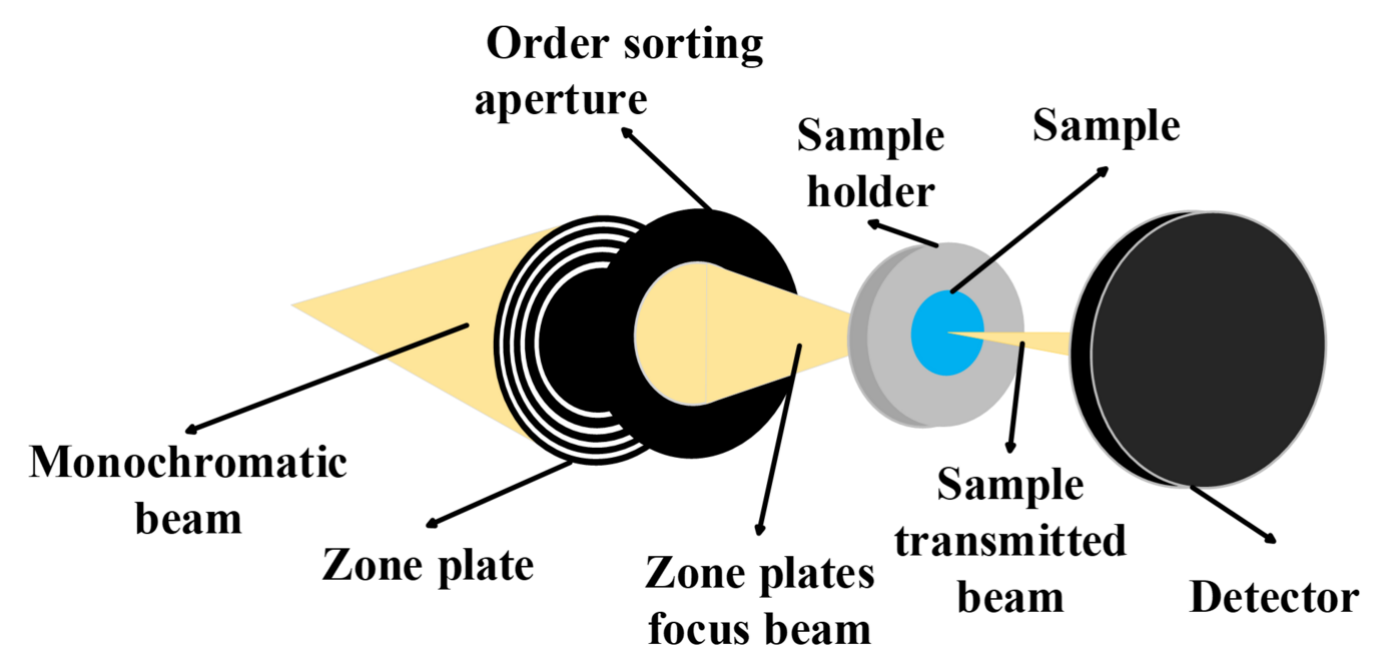
Schematic diagram of the principle of STXM.

Scanning transmission X-ray microscopy (STXM), an important synchrotron-based experimental technique (Fig. 1 ▸), provides nanoscale analysis capabilities for material and biological samples. This method employs a Fresnel zone plate and an order-sorting aperture to focus the monochromatic X-ray beam to tens of nanometres, which is then used to scan a two-dimensional sample for high-resolution imaging (Kirz *et al.*, 1992 ▸; Zhang *et al.*, 2015 ▸; Thibault *et al.*, 2008 ▸). Combining STXM with X-ray absorption spectroscopy makes it possible to map the chemical states of a sample at the nanoscale (Lewis *et al.*, 2022 ▸), facilitating detailed analysis of the chemical composition of the sample. Therefore, STXM has found extensive applications in biology, physics, materials science and environmental science (Nilsson *et al.*, 2005 ▸; Müller *et al.*, 2018 ▸; Ye *et al.*, 2016 ▸; Obst *et al.*, 2009 ▸; Vijayakumar *et al.*, 2023 ▸; Ditter *et al.*, 2022 ▸).

The detector for STXM is a photomultiplier tube (PMT) (Pilet *et al.*, 2016 ▸) which is highly sensitive to incident beam intensity fluctuations. Its discerning ability for beam intensity changes depends on the response time of the fluorescent material. If the conventional phosphor is replaced by an yttrium–aluminium–garnet (YAG):Ce crystal, the PMT can monitor the beam intensity changes at sub-microsecond scales (Gerasimova & Sinn, 2014 ▸; Bhattacharjee *et al.*, 2002 ▸), as the luminescence lifetime of a YAG:Ce crystal is approximately 100 ns (Lukyashin & Ishchenko, 2021 ▸; Hageraats *et al.*, 2021 ▸). For scanning imaging, the temporal stability of the incident beam intensity, shape and size are crucial to obtain optimal images, highlighting the significance of an efficient beam intensity monitoring system for high-quality imaging. However, the results of the STXM experiment are affected by the beam intensity stability in synchrotron radiation accelerators.

To keep beam intensity stable in synchrotron radiation accelerators, a prevalent strategy is to adopt the top-up mode in storage ring running. This running mode, which involves electron beam injection upon noticeable beam attenuation, has been employed by numerous leading synchrotron radiation facilities worldwide, such as the Stanford Synchrotron Radiation Lightsource (Bauer *et al.*, 2011 ▸), Ultraviolet Synchrotron Orbital Radiation Facility (Zen *et al.*, 2010 ▸), Super Photon Ring-8 (Asano & Takagi, 2006 ▸; Tanaka *et al.*, 2004 ▸), Taiwan Light Source (Luo *et al.*, 2007 ▸), SOLEIL (Filhol *et al.*, 2010 ▸) and the Advanced Photon Source (Emery, 2001 ▸; Emery & Borland, 2000 ▸; Emery & Borland, 2002 ▸).

The Shanghai Synchrotron Radiation Facility (SSRF) has a storage ring energy of 3.5 GeV and a maximum electron beam intensity of 220 mA (Zhao *et al.*, 2015 ▸; Barbier *et al.*, 2020 ▸). The top-up running mode (Chen *et al.*, 2019 ▸) is also employed in SSRF to maintain the storage ring current. During the early operational phase of the SSRF in the 2010s, the beam fluctuation was kept to less than 1% by injection every 10 min (Huang *et al.*, 2009 ▸). To improve the beam stability further, the facility has optimized its procedure to increase the replenishment frequency to approximately every 2 min.

**Figure 2:**
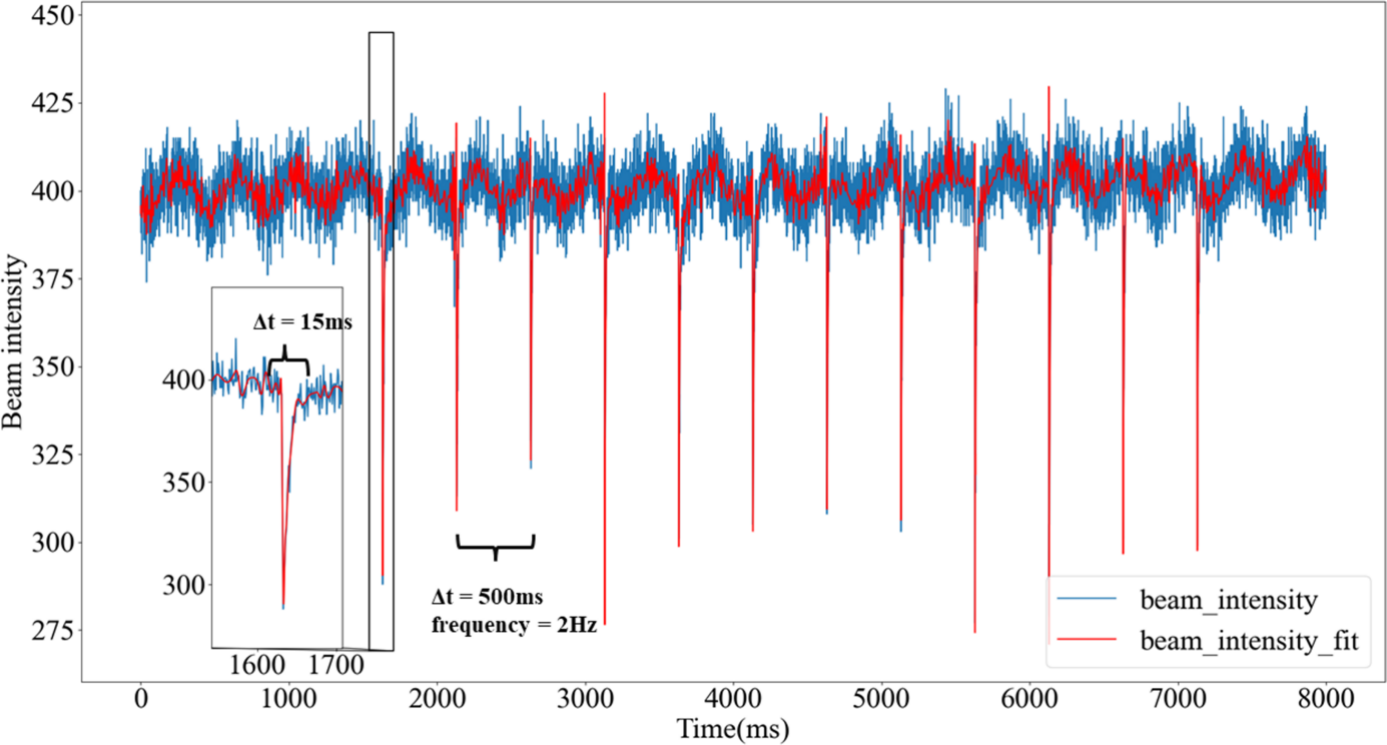
Diagram of injection-induced noise disruptions and their corresponding fitting analysis for STXM data under sample-free conditions.

During the group injection phase of the electron beam in top-up mode, discrepancies between the injection and storage beam energies led to jitters in the storage ring beam intensity. These variations induce oscillations of the stored beam, diminish/fluctuate the outgoing X-ray beam intensity and potentially impact the experimental data quality (Leng *et al.*, 2013 ▸; Yang *et al.*, 2015 ▸). In the injection phase of SSRF top-up mode, 12, 16 or more bunches are injected into the ring at a frequency of 2 bunches s^−1^. This injection process results in a 60% reduction in the soft X-ray intensity within 1 ms, which then gradually recovers in the subsequent tens of milliseconds. The STXM data collected during the electron bunch injections were analyzed and are shown in Fig. 2 ▸. It can be seen that over a period of 8 s, 12 evident noise disruptions induced by the injections were recorded, and each noise disruption lasted for approximately 15 ms. Furthermore, the interval between the noisy disruptions was estimated to be 500 ms based on the fitted data, which is consistent with the theoretical injection frequency of 2 Hz for electron bunches.

Synchrotron radiation accelerators worldwide have been exploring strategies to mitigate the effects of top-up mode on experimental data. For instance, the Infrared Micro-spectroscopy beamline at the Australian Synchrotron, which is highly susceptible to beam oscillations induced by the top-up mode, has implemented a method that halts the data acquisition during electron injections. This approach conducts the suspension and resumption of data collection based on a trigger signal from the accelerator, thereby minimizing the impact of beam fluctuations on data quality (van Garderen *et al.*, 2013 ▸). Similarly, SOLEIL’s infrared beamline SMIS employs a hardware-based solution to pause the data acquisition during top-up injections, substantially enhancing data quality and increasing the signal-to-noise ratio by 12–15 times (Ricaud *et al.*, 2013 ▸).

At the Advanced Light Source, an STXM device employs median filtering to eliminate abnormal pixels resulting from top-up injections. Although effective for anomaly removal, the accurate identification of anomalies close to the sample presents a challenge (Marcus, 2023 ▸).

However, at the SSRF, the relatively high frequency of electron beam injections poses a challenge. Pausing scans when injecting electron bunches is a possible solution to this problem but reduces the experimental throughput and disrupts the fly scan continuity. Also, achieving precise synchronization between the injection and the STXM detector pause is difficult.

Therefore, a techniques that can be used for upstream beam intensity detection is particularly necessary. Current techniques for monitoring the X-ray beam intensity at synchrotrons include using wire chambers (Qi & Liu, 2014 ▸; Bao-Guo *et al.*, 2004 ▸), ionization chambers (Ilinski *et al.*, 2007 ▸), single-crystal diamond detectors (Morgan *et al.*, 2023 ▸; Morse *et al.*, 2007 ▸; Bergonzo *et al.*, 2000 ▸) and semiconductor detectors (Feng *et al.*, 2011 ▸). However, their application in the upstream optical path of an STXM often disrupts the intensity or shape of the X-rays, thereby diminishing their utility in upstream beam intensity monitoring of STXM.

To address this challenge, an online real-time synchronous beam intensity monitoring system (BIMS) is essential for tracking intensity fluctuations during injections. Moreover, postprocessing techniques need to be developed to eliminate the impact of injection-induced intensity changes, thereby ensuring the accuracy and integrity of the experimental data. It uses a YAG crystal fixed on the upper edge of the exit slit of the beamline to interact with the incident beam and produce visible fluorescence, which is detected by a PMT detector installed close to the YAG. This PMT is identical to that used in the STXM imaging chamber. Through field-programmable gate array (FPGA)-triggered synchronization, data from both the monitoring PMT and the imaging PMT are collected simultaneously. This technique supports data acquisition rates of up to 1 MHz with a synchronization error of less than 20 ns, enabling high-speed non-intrusive real-time monitoring of beam intensity. Therefore, the technique markedly improves experimental accuracy and efficiency.

## 2. Beam intensity monitoring system: principle and design

### 2.1. Beam intensity monitoring principle

The new online BIMS is implemented at the BL08U1A beamline of the SSRF. This beamline was built to track the intensity fluctuations during injections. With the BIMS, the influence of injection-induced intensity changes on the imaging effect STXM devices has been greatly improved.

**Figure 3:**
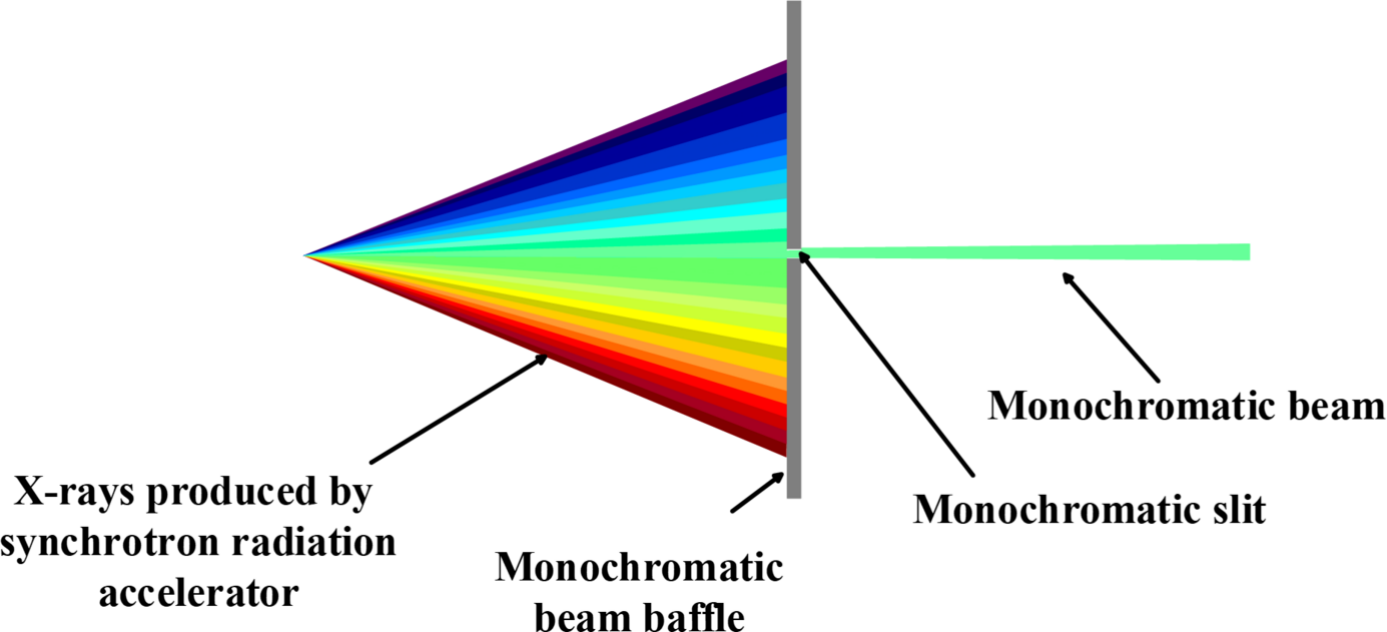
Diagram of the beamline exit slit.

Synchrotron X-rays exhibit high polarization levels (Li *et al.*, 2017 ▸) and, upon traversing a plane-grating monochromator, are dispersed into a strip of monochromated X-rays (Gong & Lu, 2015 ▸). This strip is typically approximately 300 µm in width and 15000 µm in height. As the strip of X-rays is projected onto the exit slit of the beamline, only a small fraction of the strip is allowed to pass through, leaving large portions at the upper and lower sides of the exit slit (Fig. 3 ▸), which have the same intensity variation as the transmitted X-rays. This distribution of X-rays near the slit makes it possible to use the non-transmitted segment of the X-ray beam to monitor the intensity fluctuations of the transmitted X-ray beam that enters the STXM chamber finally.

To reach this goal, a beam intensity detection module (IDM) was designed and installed at the upper side of the exit slit of the beamline. This module mirrored the detecting module of the STXM. This arrangement enabled synchronous tracking of the incident beam intensity variations for the STXM endstation without changing the shape and intensity of the incident beam.

### 2.2. Design of the beam intensity monitoring system

An STXM endstation has been built at the BL08U1A beamline of SSRF that has achieved a spatial resolution of 30 nm through zone plate focusing (Zhou *et al.*, 2011 ▸; Xu * et al.*, 2011 ▸), with a nanoscale analysis power for elements and chemical states (Xingxing *et al.*, 2011 ▸).

**Figure 4:**
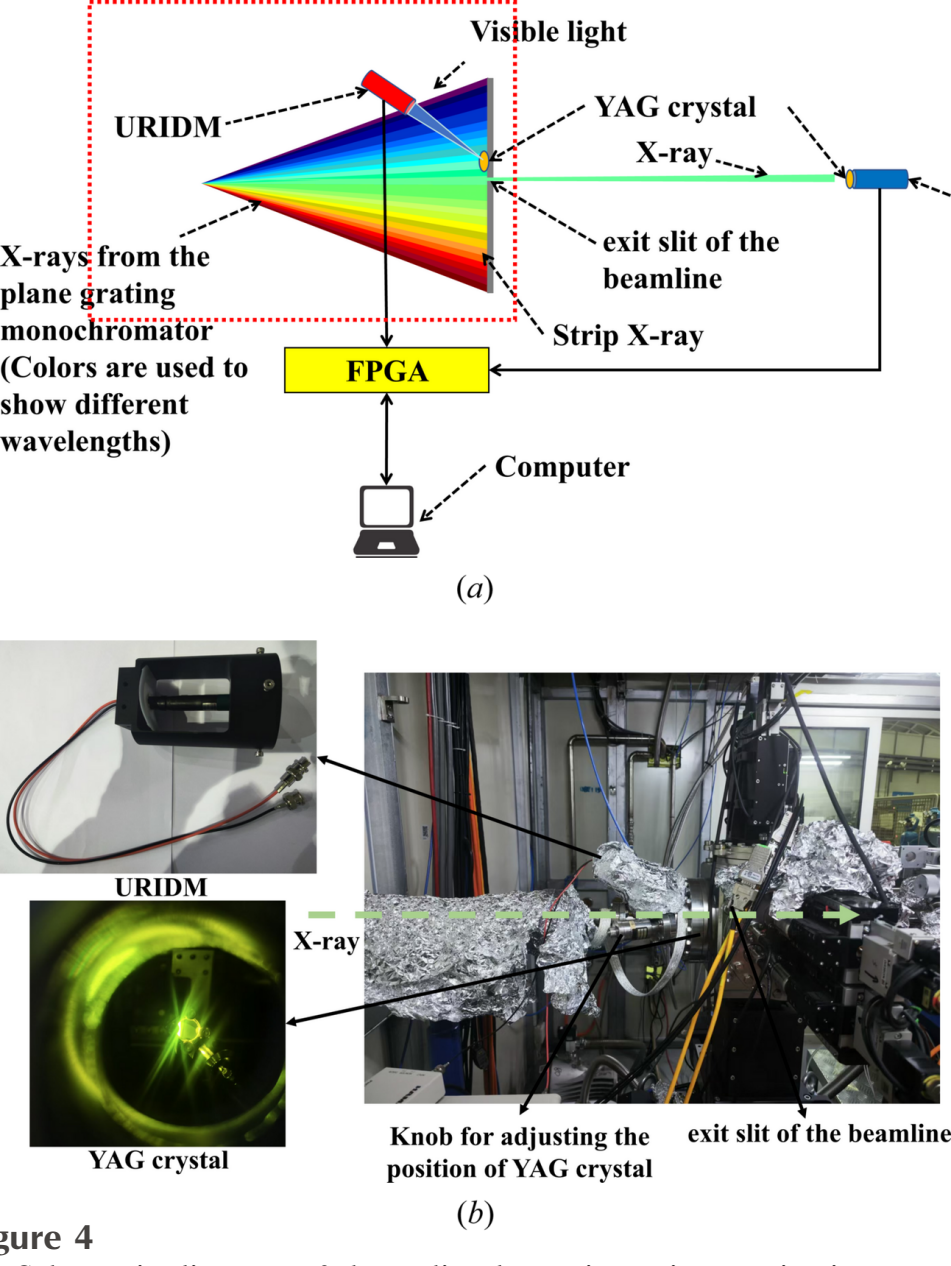
(*a*) Schematic diagram of the online beam intensity monitoring system. (*b*) Photographs of the design highlighted in the red box in (*a*) for upstream reference beam intensity monitoring.

The design of the online X-ray intensity monitoring system is shown in Fig. 4 ▸(*a*) and photographs of the design highlighted in the red dotted box in Fig. 4 ▸(*a*) for upstream reference beam intensity monitoring are shown in Fig. 4 ▸(*b*). It comprises two beam IDMs, two YAG:Ce crystals, an FPGA for triggering data acquisition, and a computer for data analysis. One of the two beam IDMs is used to monitor the upstream reference beam intensity, called the upstream reference intensity detection module (URIDM), and the other is used for STXM imaging, called the downstream imaging intensity detection module (DIIDM).

The selected YAG:Ce crystal, which is well known for its robust radiation resistance, is suitable for synchrotron radiation applications (Xu *et al.*, 2012 ▸). Its luminous lifetime of only 70 ns fully satisfies the rapid sub-millisecond data acquisition requirements of STXM scanning. Furthermore, its central emission wavelength is 550 nm which matches the spectral sensitivity range of the PMT, enabling the efficient conversion of X-rays into visible light for the PMT.

One of the two YAG crystals is at the upper side of the exit slit of the beamline, and the other is at the top of the DIIDM in the STXM chamber. This deployment does not interfere with the intensity or shape of the incident beam, thereby maintaining the integrity of the experimental conditions.

**Figure 5:**
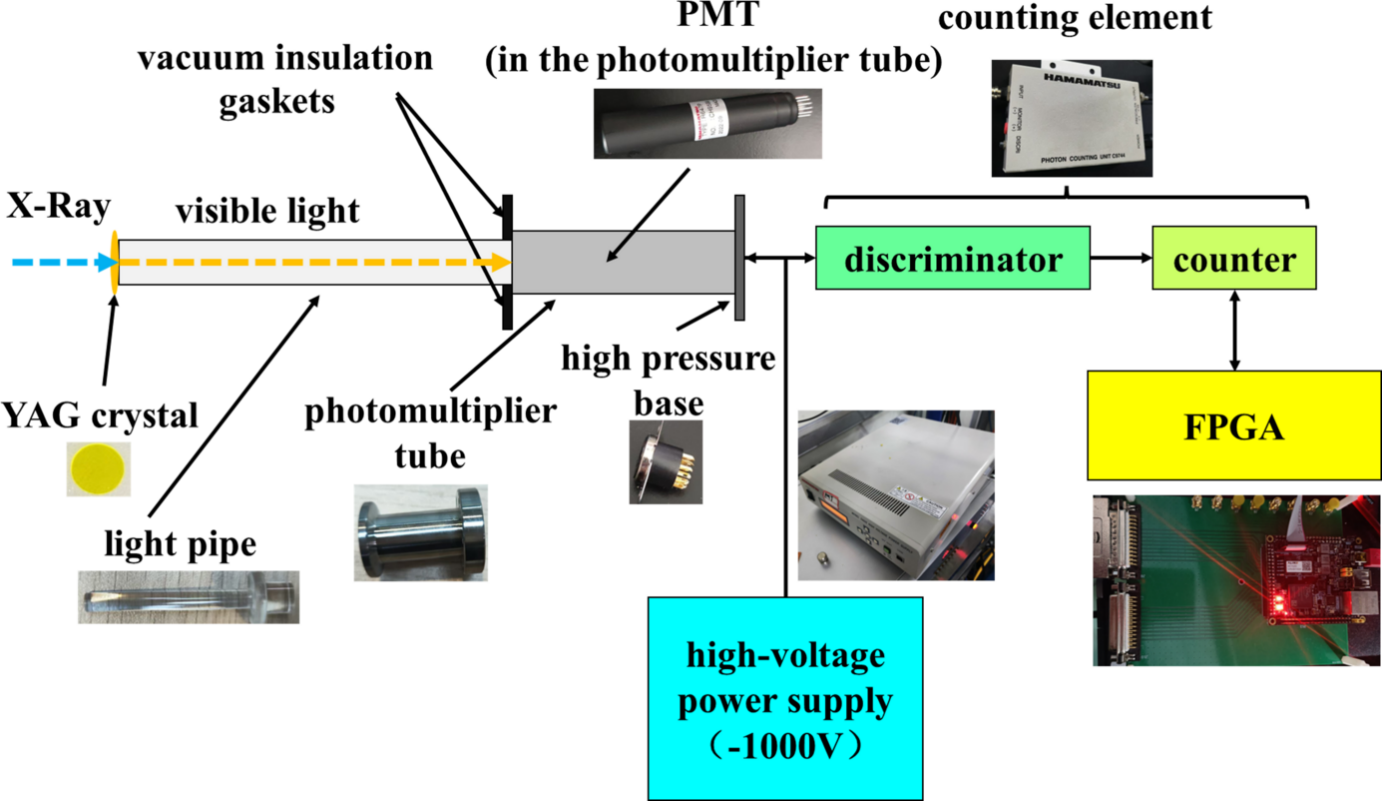
Diagram of the beam intensity detecting module.

The IDM depicted in Fig. 5 ▸ includes a photon light pipe, PMT, high-voltage power supply, and a counting element (Herbert *et al.*, 2006 ▸; Ting-Feng, 2009 ▸). The photon light pipe gathers visible light emitted from the YAG crystal while filtering out any stray beam. Given the soft X-rays used in STXM, which requires vacuum conditions to avoid X-ray absorption by air, a vacuum insulation gasket is placed between the light pipe and the PMT to maintain the vacuum environment. The selected PMT, operating at a high voltage of 1000 V, has a wavelength detection range of 300–650 nm, accommodating the 550 nm-wavelength visible light from the YAG crystal. The model number for the PMT is R647P and that for the high-voltage power supply is C9525-03.

The counting component of the module includes a discriminator for signal shaping and a counter for photon tallying. The discriminator has been integrated into the photon counter, model number C9744. This counter interfaces with an FPGA development board through an SMA-BNC signal trigger line. The model number of the FPGA is ZYNQ XA72020. The FPGA, with a 125 MHz clock frequency, facilitates high-speed data acquisition at 1 MHz and enables simultaneous counting of the two IDMs with a 100 MHz clock frequency. This setup has a maximum trigger time difference of 20 ns between the two IDMs, which is less than the luminous lifetime of the YAG crystal and significantly less than the 1 ms acquisition time usually used in STXM imaging, ensuring the precision and efficiency of the beam intensity detecting. The thicknesses of the two YAG crystals are 0.5 mm. The diameters of the YAG crystals of the URIDM are 10 mm and the size of the YAG crystals of the DIIDM is 5 mm × 5 mm.

### 2.3. Integration of the system into the STXM endstation

**Figure 6:**
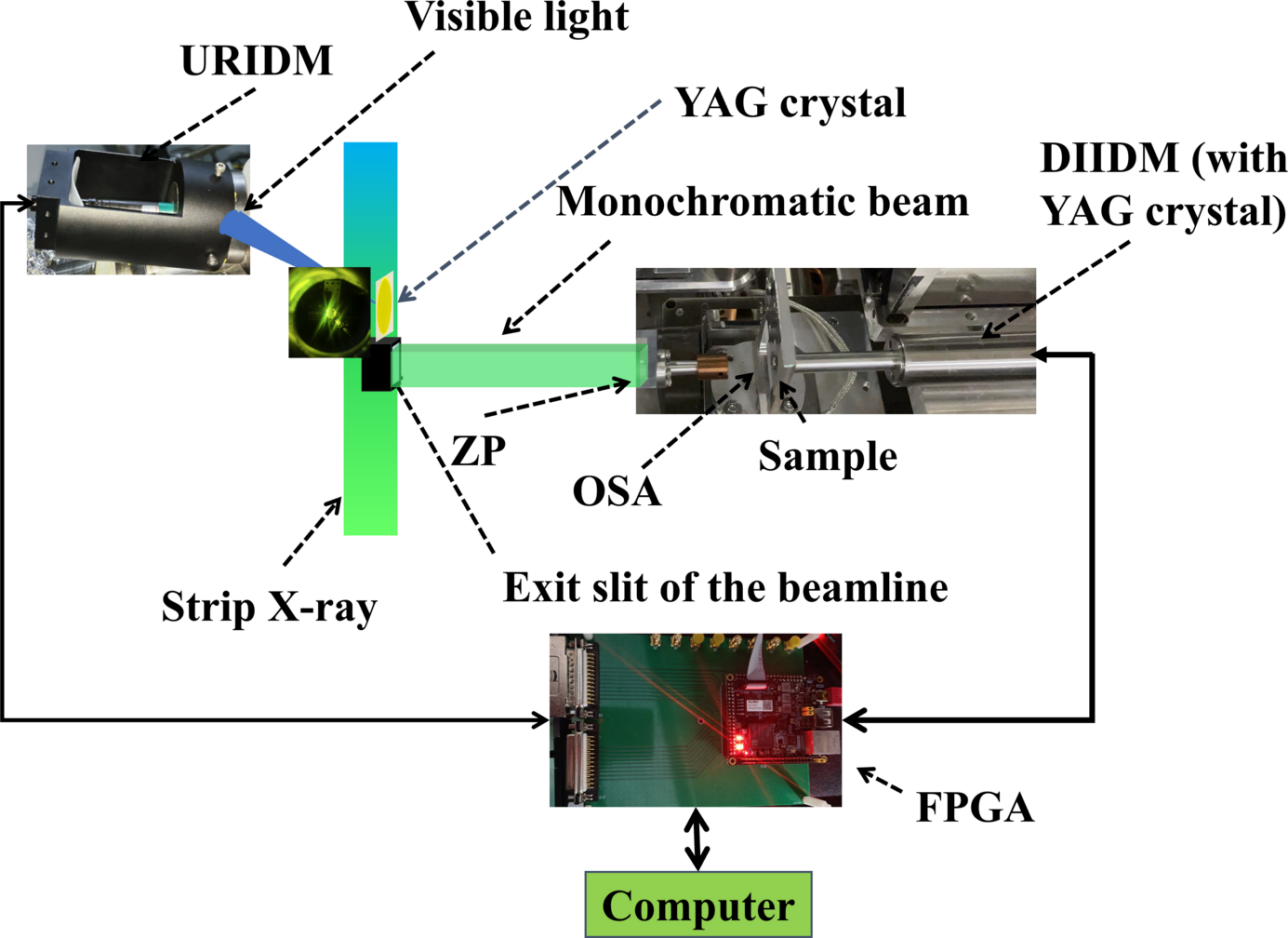
Application of the online beam intensity monitoring system in STXM.

The configuration of the system within the STXM station is shown in Fig. 6 ▸. Two IDMs are positioned upstream of the exit slit of the beamline and downstream of the sample holder, respectively, to measure the incoming and transmitted X-ray intensity through the sample. The PMT detectors in both IDMs are coordinated by FPGA-controlled commands to synchronize the data acquisition. The data processing is conducted on a computer utilizing the synchronously recorded beam intensity data from the two IDMs during the STXM experiment process.

The dual IDMs of the system, using identical YAG crystals and PMT detectors, ensure the synchronization between tracking the beam intensity fluctuations and recording the STXM signals. Both modules, powered by an FPGA and two photon counters with an extremely high clock frequency, can achieve a remarkably fast synchronous acquisition rate. Under the FPGA triggering, a time lag of only 20 ns exists between the two PMT detectors. Consequently, the reference beam intensity variations captured by the URIDM align well in time with the transmitted intensity recorded by the DIIDM. This synchronization allows for the correction of the transmitted intensity signals of the STXM based on the URIDM data. This online BIMS provides a solution to the issue of beam intensity noise resulting from the electron beam injection through image postprocessing. This capability ensures the experimental efficiency and data integrity of the STXM endstation.

**Figure 7:**
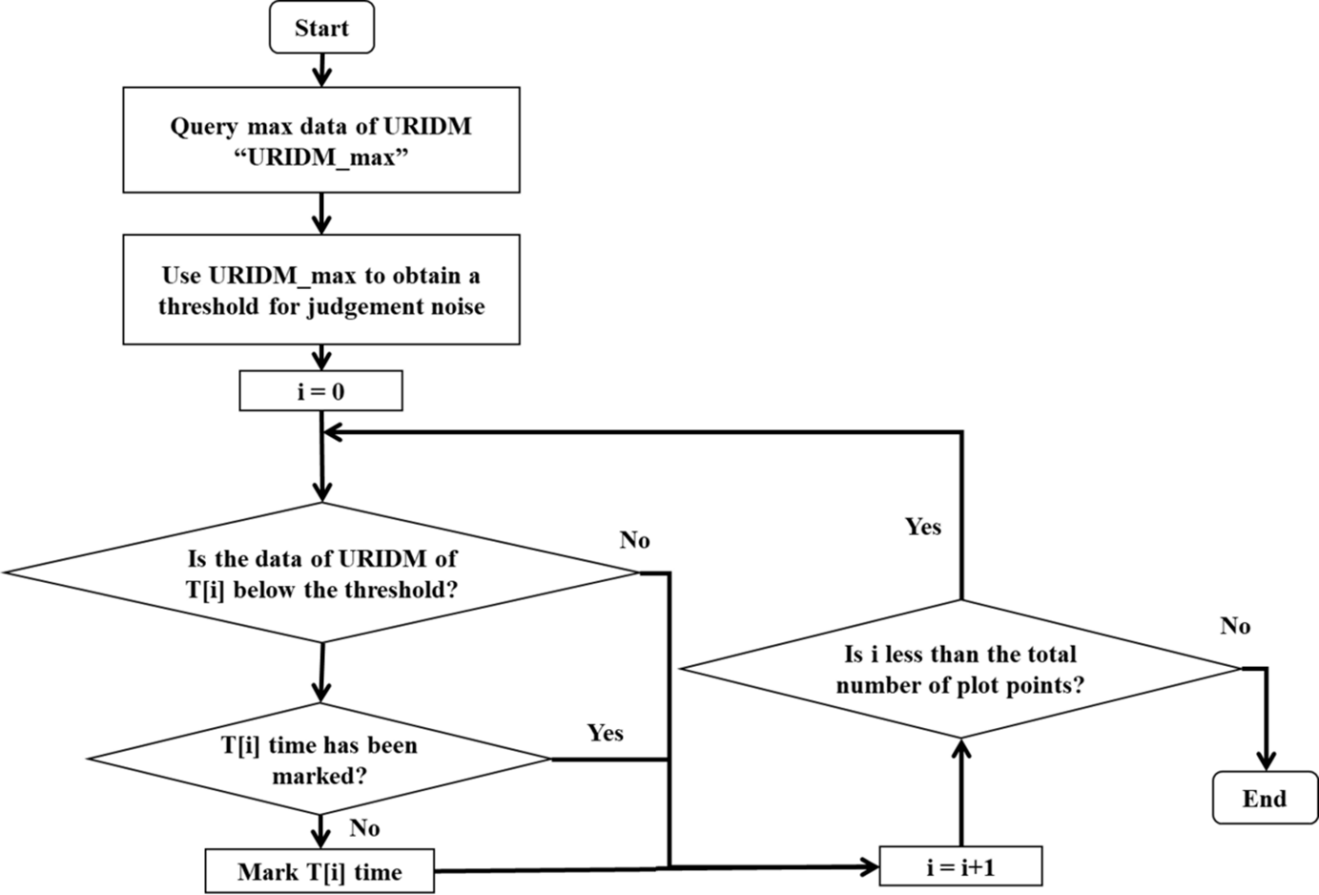
Flowchart of the data postprocessing algorithm to identify the injection-induced beam intensity drop positions and remove the corresponding noise points from the STXM image.

An image postprocessing algorithm was designed together with the BIMS to remove the injection-induced noise from the STXM images by using the incident intensity data of the BIMS. Its flowchart is shown in Fig. 7 ▸. In this process, firstly the beam intensity sharp-drop points in the data recorded by the URIDM are identified through manually setting a threshold. These identified points in the incident beam are then used to precisely locate the corresponding noise points in the STXM data. These noise points in the STXM image are then filtered out by averaging. This approach effectively eliminates the adverse effects of the beam intensity sharp-drops induced by electron beam injections, and ensures the quality of the STXM image.

## 3. Main performance test and discussion

### 3.1. Monitoring beam intensity change trend

**Figure 8:**
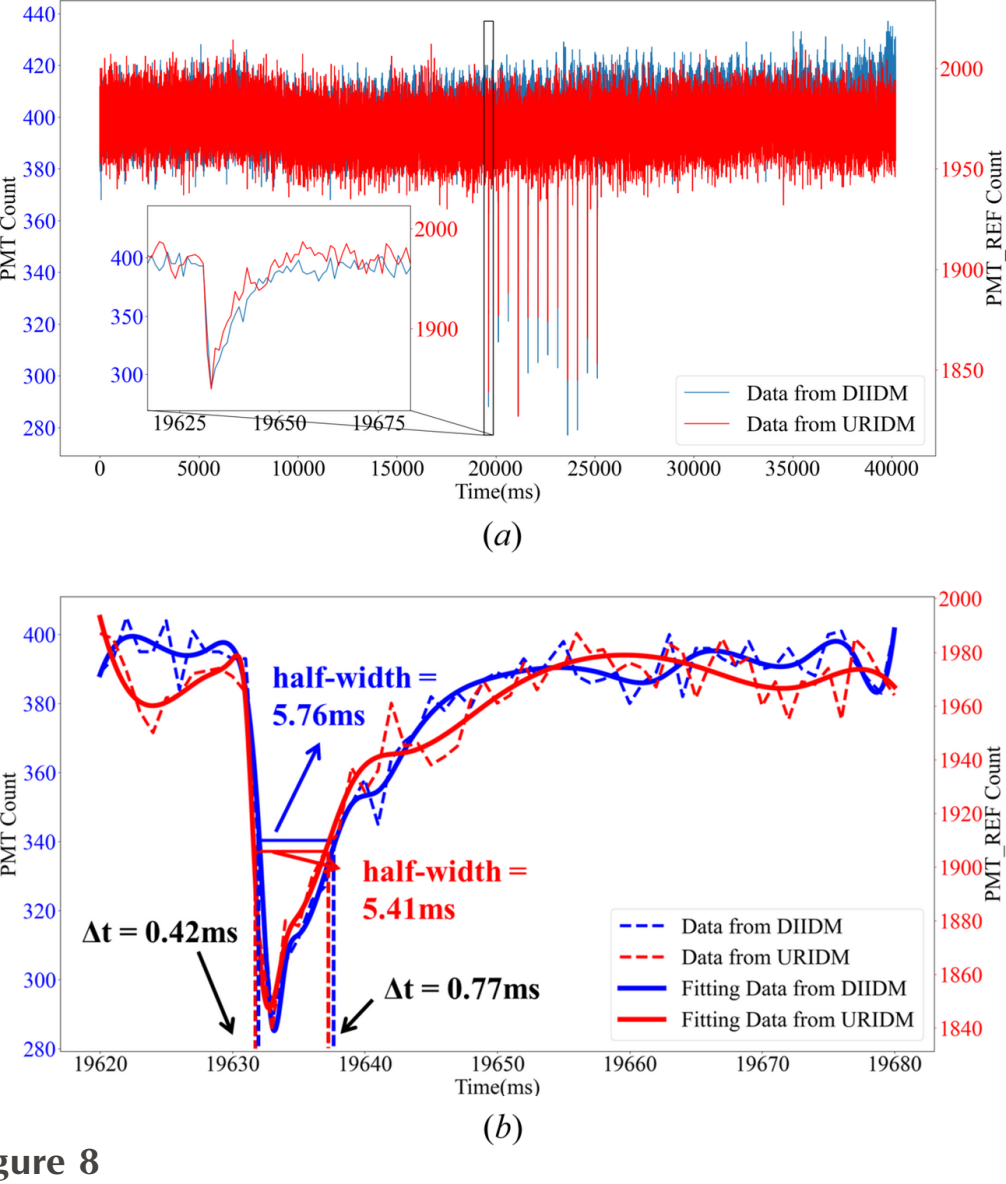
The BIMS collected 40000 data points at a 1 kHz frequency to validate the synchronization between the two IDMs. The red curve represents data from the URIDM, while the blue curve shows the measurement from the DIIDM. (*a*) The originally collected data and (*b*) the data fitting and analysis of the magnified section in (*a*), showing the high synchronicity between the two IDMs.

A beam intensity change monitoring experiment was conducted at the STXM beamline of the SSRF. In this experiment, the STXM scans were performed over a uniform, sample-free area to capture the direct beam data. By chronologically organizing the 40000 data points collected at 1 kHz rate, the image shown in Fig. 8 ▸(*a*) was generated. The red curve represents the reference beam intensity recorded by the URIDM, and the blue curve shows the transmitted beam intensity recorded by the DIIDM. The image reveals a series of 12 pronounced beam intensity drops, spaced 0.5 s apart, corresponding to the 12 electron bunch injections at a frequency of 2 Hz.

Fig. 8 ▸(*b*) magnifies a small segment of the curves in Fig. 8 ▸(*a*), illustrating that the beam intensity change trends measured by both of the IDMs are virtually identical and demonstrating a precise synchronization.

In Fig. 8 ▸(*b*), curve fitting was applied to the magnified segment from Fig. 8 ▸(*a*), making it possible to calculate the full width at half-maximum (FWHM) heights of the two valley curves while the beam intensity sharply drops induced by electron beam injections. A comparative analysis of the fitted data revealed that the FWHMs and valley-bottom time points of both curves aligned closely. Δ*t* in the figure was used for the time difference between the two endpoint moments of the corresponding FWHM of the two curves. This observation indicates the high degree of synchronization between the BIMS and the STXM detector, and demonstrates the efficacy of the system for precisely determining the injection timing of the electron bunches.

**Figure 9:**
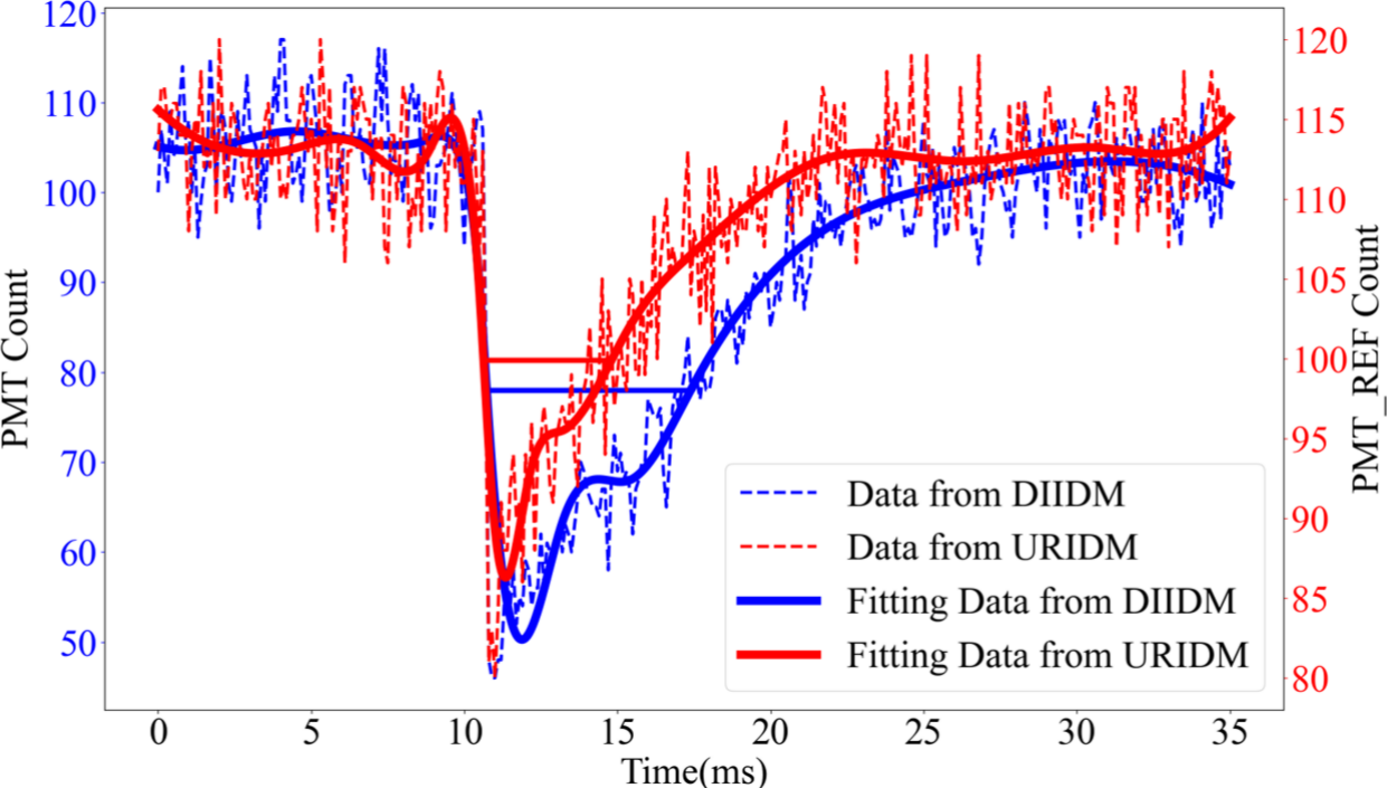
A segment of 3500 data points around the time point of an electron bunch injection were collected by the BIMS at a 10 kHz acquisition frequency. This detailed view further confirms the accurate synchronization between the IDMs. The red dotted line depicts the reference beam intensity data from the URIDM, while the blue dotted line depicts the transmitted intensity data from the DIIDM. The red and blue solid curves represent the data-fitting results of the two dotted lines, respectively, demonstrating the precision and coordination of the BIMS in capturing beam intensity fluctuations.

To further verify the synchronization of the two sets of IDMs, 400000 data points were captured at a 10 kHz frequency, and plotted chronologically. By focusing on the data collected within the time interval of 35 ms around an electron bunch injection, the image shown in Fig. 9 ▸ was generated. The red dotted line illustrates the reference beam intensity recorded by the URIDM, and the blue dotted line shows the transmitted intensity recorded by the DIIDM in the STXM chamber.

The concurrent descent observed in the red and blue curves confirms the capability of the online BIMS to track electron beam injection signals accurately with a 100 µs time resolution. This high level of synchronization between the two IDMs satisfies the requirements of the STXM experiment for millisecond sampling and high-speed monitoring of beam intensity fluctuations.

### 3.2. STXM data correction using the BIMS

**Figure 10:**
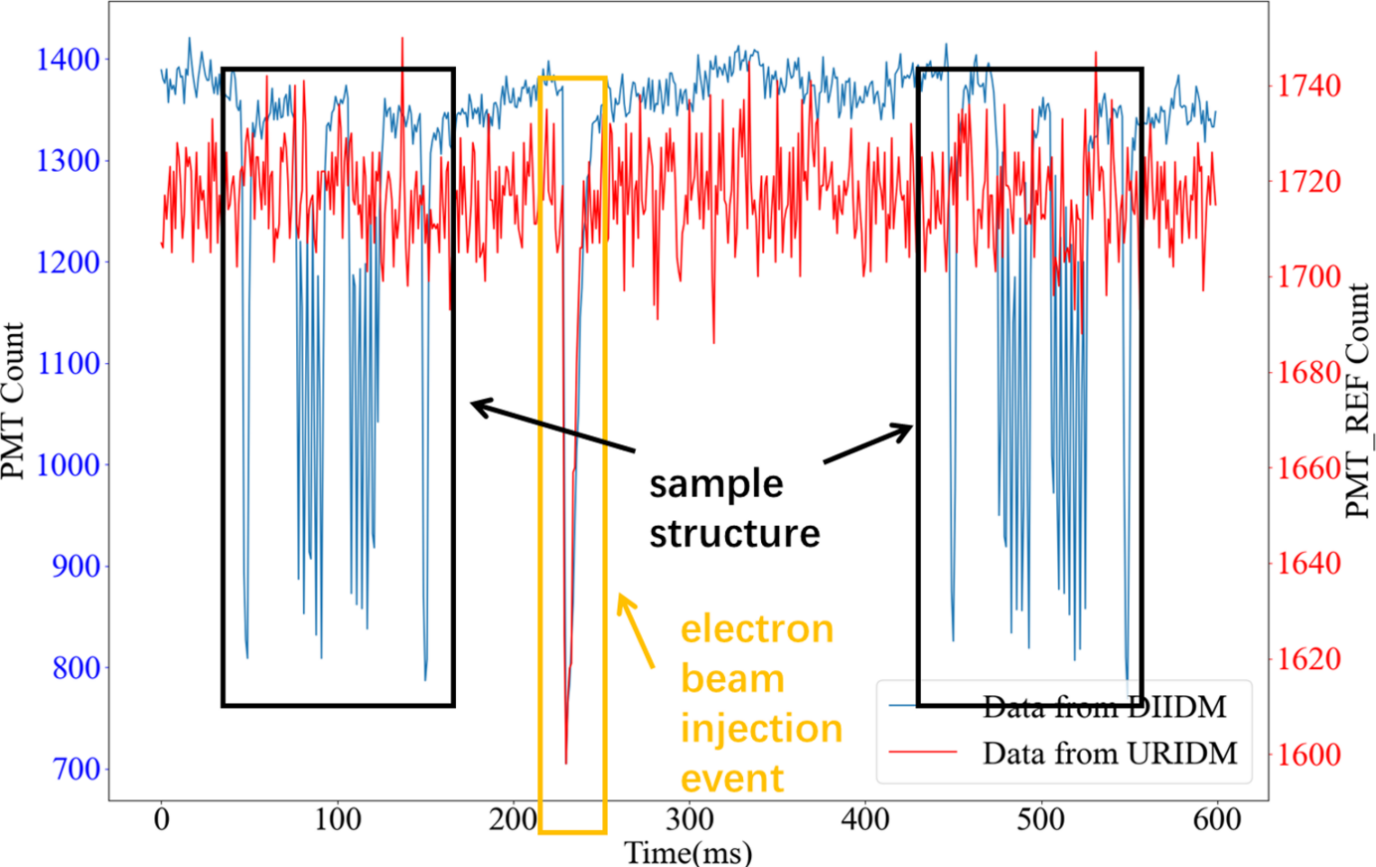
A segment of 600 data points from both the URIDM and the DIIDM following the STXM data acquisition of a non-uniform sample at a 1 kHz frequency. The data involve sample structures and an electron beam injection event. The two curves were analyzed to scrutinize the synchronization and temporal resolution of the two IDMs. The red curve displays the reference beam intensity data captured by the URIDM, whereas the blue curve displays the transmitted intensity data collected by the DIIDM, indicating the precision and coordination of the BIMS with the STXM imaging.

To evaluate the effectiveness of the beam intensity monitoring approach as well as the data postprocessing algorithm, an STXM experiment was conducted to collect 600 data points at a 1 kHz frequency. These data points were organized chronologically, involving an electron bunch injection event and some sample structure responses, as shown in Fig. 10 ▸. The blue curve represents the transmitted intensity values from the DIIDM, whereas the red curve corresponds to the values from the URIDM. Synchronous dips in both curves indicate the occurrence of an electron beam injection, whereas the drops in the blue curve are imaging signals resulting from sample absorption. This analysis confirmed the ability of the approach to differentiate accurately the sample-absorption-induced beam intensity changes from the ones induced by electron beam injections of the synchrotron accelerator.

To confirm the effectiveness of the BIMS in tracking the injection-induced beam intensity drop and improving imaging results, another STXM scan was conducted on the sample with the real-time incident intensity recorded by the online synchronous BIMS.

**Figure 11:**
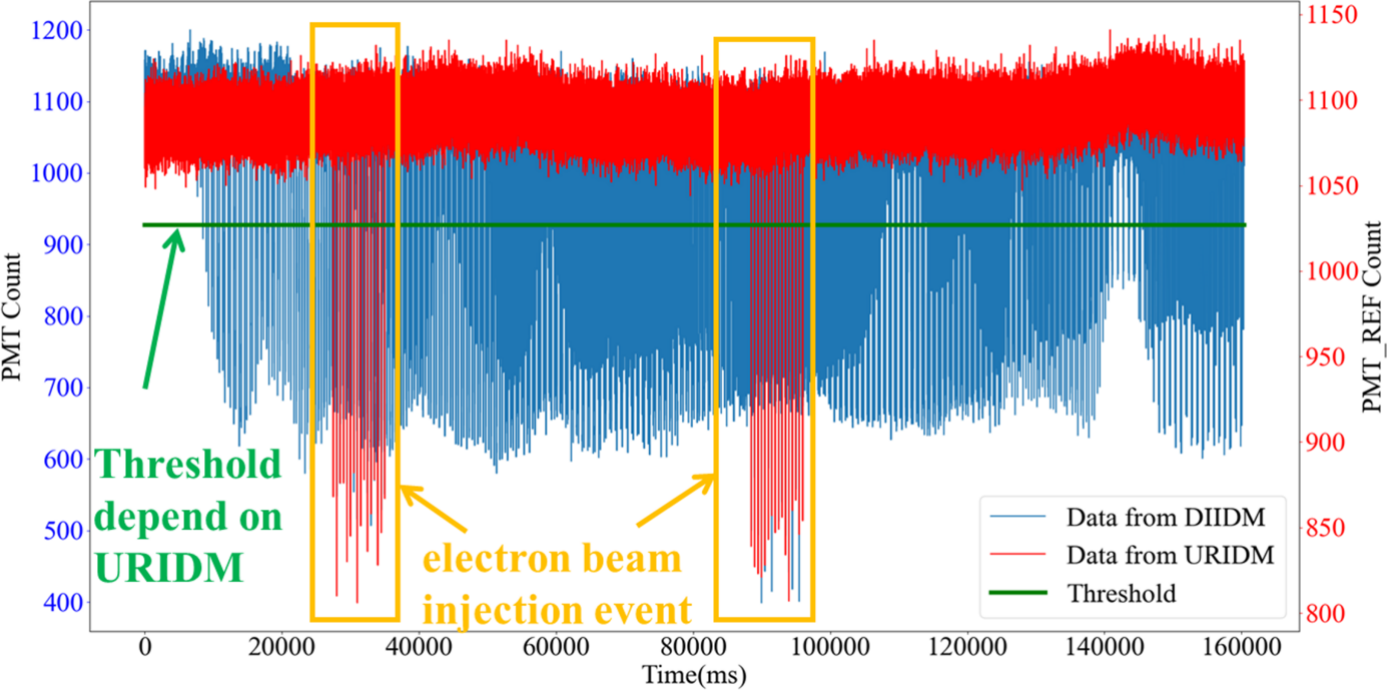
Two datasets, each containing 160000 data points, were acquired at a 1 kHz frequency by the URIDM and the DIIDM, respectively, following an STXM scan of a sample. The data are sequenced chronologically. The red curve represents the reference intensity data captured by the URIDM, while the blue curve represents the imaging data captured by the DIIDM, providing an overview of beam intensity fluctuations over time.

With a 1 kHz sampling frequency, a dataset of 160000 points was amassed, shown chronologically in Fig. 11 ▸. The red curve represents the reference beam intensity captured by the URIDM, whereas the blue curve represents the transmitted intensity recorded by the DIIDM within the STXM chamber. During electron beam injections, the red and blue curves exhibit simultaneous declines. Conversely, in instances featuring the sample structure, only the blue curve demonstrates variations. Hence, the reference beam intensity fluctuations captured by the URIDM offer a basis (the green line in Fig. 11 ▸) for positioning the time points of the electron beam injections, which can be used to denoise the sample STXM images without decreasing the imaging quality.

**Figure 12:**
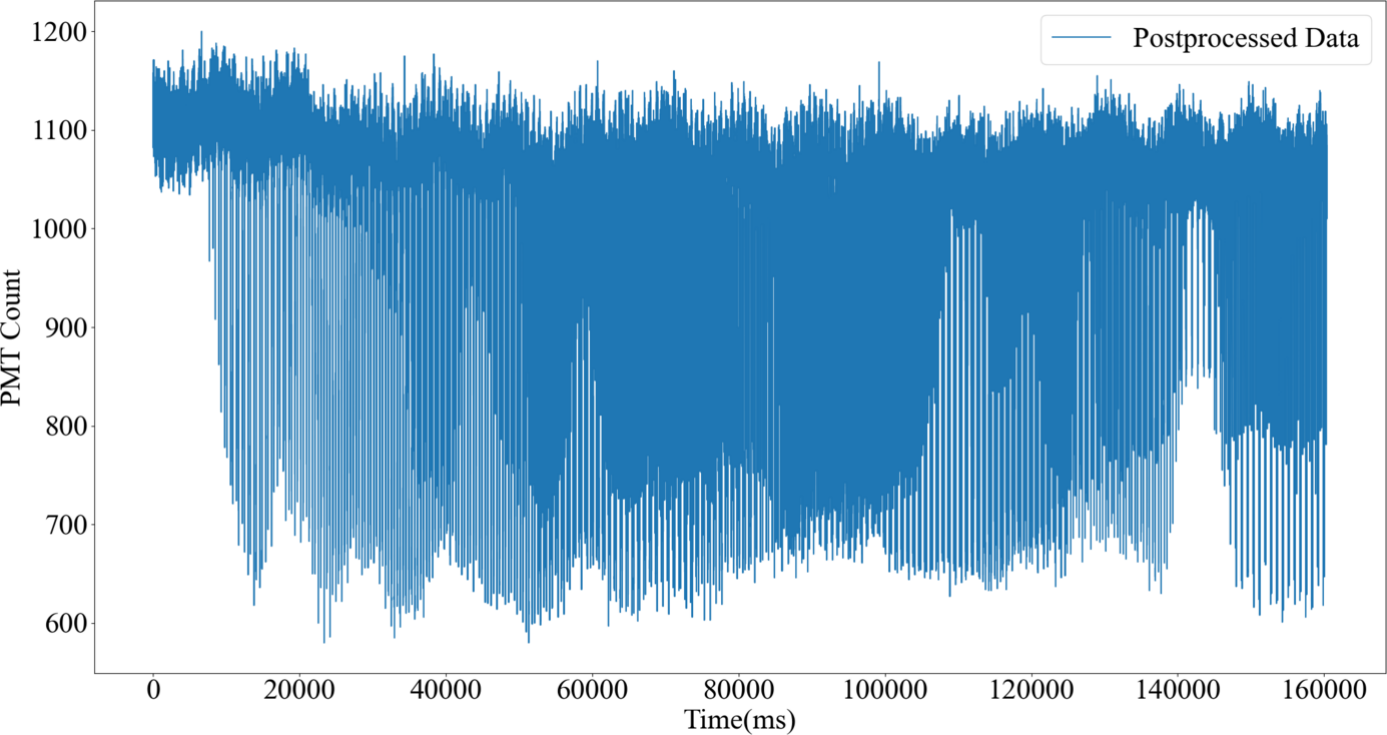
The STXM data from Fig. 11 ▸ are postprocessed using the reference beam intensity data recorded by the URIDM. Sharp-drop points or noise points owing to the electron beam injections are removed from the STXM data.

Based on the injection-induced beam intensity droppings shown in Fig. 11 ▸, the incident intensity changes can be used to pinpoint the moments of electron beam injections, and mean filtering can be applied to smooth these intensity sharp drops in the STXM image. The resultant STXM image (Fig. 12 ▸) shows that the synchronous declines observed in the red and blue curves of Fig. 11 ▸ were greatly mitigated or removed in Fig. 12 ▸, while the segments where only the blue curve drops in Fig. 11 ▸ remained unchanged in Fig. 12 ▸. This result demonstrates the ability of the synchronous BIMS to remove the injection-induced noise in STXM images without decreasing the STXM resolution.

**Figure 13:**
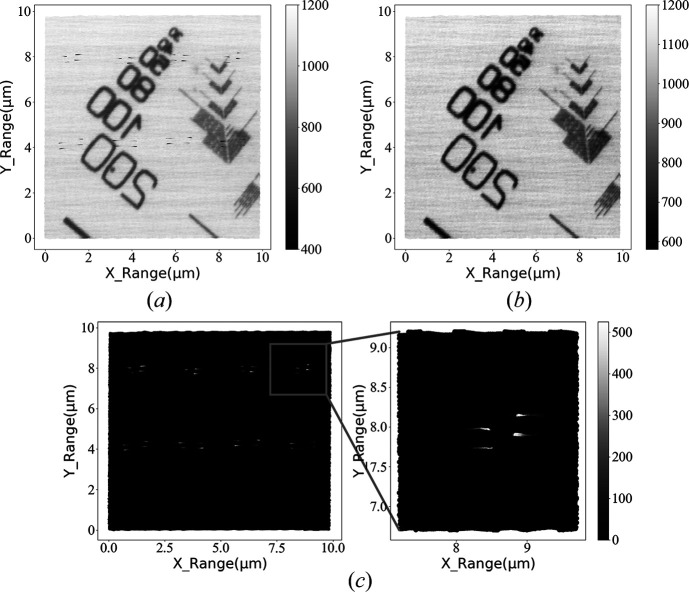
(*a*) STXM image without denoise processing, featuring two groups of noise spots resulting from two rounds of electron beam injections. (*b*) Through identifying and positioning the electron beam injection events using the data captured by the URIDM of the BIMS, the noise spots in the STXM image are removed by the developed image postprocessing algorithm. This process eliminates the impacts of fluctuating beam intensity and electron beam injection on the STXM imaging quality. (*c*) The left image is obtained by subtracting image (*b*) from (*a*) to illustrate the effect of electron beam injection events. The right image is a zoom of a part of the left image with electron beam injection events.

To directly display the denoising capability of the BIMS with the image postprocessing algorithm, a 10 µm × 10 µm sample area was imaged at the STXM endstation at a 1 kHz sampling rate. A total of 160000 data points were collected along with their corresponding spatial locations to produce the image shown in Fig. 13 ▸(*a*). The image shows that during the STXM scan the synchrotron accelerator performed two rounds of injections, resulting in 32 intensity sharp-drop points. These sharp-drop points lead to black noise spots in the STXM image, which decrease the image contrast and significantly impair the image quality.

After applying the image postprocessing algorithm to the original image [Fig. 13 ▸(*a*)], the denoised image was obtained and is shown in Fig. 13 ▸(*b*). We can see that the black spots caused by the electron bunch injections have been removed completely, and the sample information is almost unaffected by the processing. In addition, the overall uniformity of the image has been significantly improved compared with Fig. 13 ▸(*a*). Fig. 13 ▸(*c*) shows the result of Fig. 13 ▸(*b*) minus Fig. 13 ▸(*a*) for highlighting the eliminated injection-induced noise.

The effectiveness of elemental analysis through energy stack imaging of the sample around the absorption edge in STXM can be heavily reduced by the incident beam intensity variations during imaging. Implementing this online synchronous BIMS not only enhances the uniformity of the image contrast but also greatly mitigates the impact of X-ray intensity fluctuations due to the storage ring instability on the imaging quality.

## 4. Conclusion

An online synchronous beam intensity monitoring system was developed, which can significantly enhance the ability of an STXM beamline to track the incident beam intensity fluctuations and improve the imaging performance of the STXM endstation. With this system, the real-time monitoring of the beam intensity drop caused by electron bunch injections of the synchrotron accelerator is achieved without changing the shape and intensity of the incident beam on the sample. By synchronizing two IDMs with 20 ns accuracy and supporting an acquisition rate of up to 1 MHz, this system ensures rapid collection of reference beam intensity data synchronized with STXM imaging. By postprocessing the reference intensity data together with the STXM imaging data, the adverse effects of the electron beam injections on the imaging quality can be eliminated, thereby substantially improving the STXM image quality.

In addition, the online BIMS is especially important for long-period experiments such as energy stack imaging, which are particularly sensitive to the beam intensity variations over long periods of time. Therefore, the developed BIMS also significantly improves the elemental analysis quality of long-period experiments.

## References

[bb1] Asano, Y. & Takagi, T. (2006). *Radiat. Meas.* **41**, S236–S241.

[bb2] Bao-Guo, Z., Wan-Xin, W., Zhi-Yong, W. *et al.* (2004). *At. Energy Sci. Technol.* **38**, 459–462.

[bb3] Barbier, A., Xu, J., Cui, J., Machefel, A., Mantileri, C., Maccagnan, M., Rogez, E. & Roig, M. (2020). *IOP Conf. Ser. Mater. Sci. Eng.* **755**, 012083.

[bb4] Bauer, J. M., Liu, J. C., Prinz, A. A. & Rokni, S. H. (2011). *Nucl. Technol.* **175**, 198–201.

[bb5] Bergonzo, P., Brambilla, A., Tromson, D., Mer, C., Hordequin, C., Guizard, B., Foulon, F., Solé, V. A. & Gauthier, C. (2000). *Diamond Relat. Mater.* **9**, 960–964.

[bb6] Bhattacharjee, T., Basu, S. K., Dey, C. C. & Chatterjee, M. B. (2002). *Nucl. Instrum. Methods Phys. Res. A*, **484**, 364–368.

[bb7] Chen, F., Chen, Z., Zhou, Y., *et al.* (2019). *Nucl. Sci. Tech.* **30**, 3–10.

[bb8] Ditter, A. S., Smiles, D. E., Lussier, D., Altman, A. B., Bachhav, M., He, L., Mara, M. W., Degueldre, C., Minasian, S. G. & Shuh, D. K. (2022). *J. Synchrotron Rad.* **29**, 67–79. 10.1107/S1600577521012315 PMC8733983 34985424

[bb9] Emery, L. (2001). *Proceedings of the 2001 Particle Accelerator Conference (PAC2001)*, 18–22 June 2001, Chicago, IL, USA.

[bb10] Emery, L. & Borland, M. (2000). *AIP Conf. Proc.* **521**, 409–414.

[bb11] Emery, L. & Borland, M. (2002). *Proceedings of the 8th European Particle Accelerator Conference (EPAC2002)*, 3–7 June 2002, Paris, France, pp. 218–220.

[bb12] Feng, Y., Feldkamp, J. M., Fritz, D. M., Cammarata, M., Aymeric, R., Caronna, C., Lemke, H. T., Zhu, D., Lee, S., Boutet, S., Williams, G., Tono, K., Yabashi, M. & Hastings, J. B. (2011). *Proc. SPIE*, **8140**, 81400Q.

[bb13] Filhol, J. M., Nadji, A., Besson, J. C., *et al.* (2010). *Proceedings of the 1st International Particle Accelerator Conference (IPAC2010)*, 23–28 May 2010, Kyoto, Japan.

[bb38] Garderen, E. D. van, Bambery, K. R., Clift, M., LeBlanc, G. S., Martin, D. E., Puskar, L., Starritt, A., Tobin, M. J., Wang, D. & Zhu, D. (2013). *J. Phys. Conf. Ser.* **425**, 042015.

[bb14] Gerasimova, N. & Sinn, H. (2014). *Proceedings of the 36th International Free-Electron Laser Conference (FEL2014)*, 25–29 August 2014, Basel, Switzerland.

[bb15] Gong, X. & Lu, Q. (2015). *J. X-ray Sci. Technol.* **23**, 409–421. 10.3233/XST-150497 26410653

[bb16] Hageraats, S., Keune, K., Stanescu, S., Laurent, J.-M., Fresquet, W. & Thoury, M. (2021). *J. Synchrotron Rad.* **28**, 1858–1864. 10.1107/S1600577521009450 PMC8570211 34738940

[bb17] Herbert, D. J., Saveliev, V., Belcari, N., D’Ascenzo, N., Del Guerra, A. & Golovin, A. (2006). *IEEE Trans. Nucl. Sci.* **53**, 389–394.

[bb18] Huang, S., Yan, Y. & Leng, Y. (2009). *Nucl. Sci. Tech.* **20**, 71–75.

[bb19] Ilinski, P., Hahn, U., Schulte-Schrepping, H. & Degenhardt, M. (2007). *AIP Conf. Proc.* **879**, 782–785.

[bb20] Kirz, J., Ade, H., Jacobsen, C., Ko, C., Lindaas, S., McNulty, I., Sayre, D., Williams, S., Zhang, X. & Howells, M. (1992). *Rev. Sci. Instrum.* **63**, 557–563.

[bb21] Leng, Y. B., Yang, Y., Zhang, N., *et al.* (2013). *Proceedings of IBIC2013.**Proceedings of the 2nd International Beam Instrumentation Conference (IBIC2013)*, 16–19 September 2013, Oxford, UK.

[bb22] Lewis, S. L., Russell, L. M., Saliba, G., Quinn, P. K., Bates, T. S., Carlson, C. A., Baetge, N., Aluwihare, L. I., Boss, E., Frossard, A. A., Bell, T. G. & Behrenfeld, M. J. (2022). *ACS Earth Space Chem.* **6**, 1899–1913.

[bb23] Li, C., Zhang, Q., Zhang, M., *et al.* (2017). *Proceedings of the 8th International Particle Accelerator Conference (IPAC2017)*, 14–19 May 2017, Copenhagen, Denmark.

[bb24] Lukyashin, K. E. & Ishchenko, A. V. (2021). *Russ. J. Inorg. Chem.* **66**, 1203–1211.

[bb25] Luo, G. H., Chang, H. P., Chang, J. C., Chen, C. T., Chen, J., Chen, J. R., Fann, C. S., Hsu, K. T., Hwang, C. S., Kuo, C. C., Liu, K. B., Liu, Y. C., Sheu, R. J., Ueng, T. S., Wang, D. J. & Wang, M. H. (2007). *AIP Conf. Proc.* **879**, 13–16.

[bb26] Marcus, M. A. (2023). *J. Electron Spectrosc. Relat. Phenom.* **264**, 147310.

[bb27] Morgan, A. F. D., Thomas, C. (2023). *Proceedings of the 10th European Workshop on Beam Diagnostics and Instrumentation for Particle Accelerators (DIPAC2011)*, 16–18 May 2011, Hamburg, Germany.

[bb28] Morse, J., Salomé, M., Berdermann, E., Pomorski, M., Cunningham, W. & Grant, J. (2007). *Diamond Relat. Mater.* **16**, 1049–1052.

[bb29] Müller, A., Swaraj, S., Sparnacci, K. & Unger, W. E. S. (2018). *Surf. Interface Anal.* **50**, 1077–1082.

[bb30] Nilsson, H. J., Tyliszczak, T., Wilson, R. E., Werme, L. & Shuh, D. K. (2005). *Anal. Bioanal. Chem.* **383**, 41–47. 10.1007/s00216-005-3355-5 16021423

[bb31] Obst, M., Dynes, J. J., Lawrence, J. R., Swerhone, G. D. W., Benzerara, K., Karunakaran, C., Kaznatcheev, K., Tyliszczak, T. & Hitchcock, A. P. (2009). *Geochim. Cosmochim. Acta*, **73**, 4180–4198.

[bb32] Pilet, N., Lisunova, Y., Lamattina, F., Stevenson, S. E., Pigozzi, G., Paruch, P., Fink, R. H., Hug, H. J., Quitmann, C. & Raabe, J. (2016). *Nanotechnology*, **27**, 235705. 10.1088/0957-4484/27/23/235705 27146329

[bb33] Qi, H. & Liu, M. (2014). *Int. J. Mod. Phys. Conf. Ser.* **27**, 1460142.

[bb34] Ricaud, J. P., Cassinari, L., Dumas, P., *et al.* (2013). *Proceedings of IBIC2013.**Proceedings of the 2nd International Beam Instrumentation Conference (IBIC2013)*, 16–19 September 2013, Oxford, UK.

[bb35] Tanaka, H., Aoki, T., Asaka, T., *et al.* (2004). *Proceedings of the 9th Particle Accelerator Conference (EPAC2004)*, 5–7 July 2004, Lucerne, Switzerland.

[bb36] Thibault, P., Dierolf, M., Menzel, A., Bunk, O., David, C. & Pfeiffer, F. (2008). *Science*, **321**, 379–382. 10.1126/science.1158573 18635796

[bb37] Ting-Feng, W. (2009). *OME Inf.* **2**, 39–44.

[bb39] Vijayakumar, J., Yuan, H., Mille, N., Stanescu, S., Swaraj, S., Favre-Nicolin, V., Najafi, E., Hitchcock, A. P. & Belkhou, R. (2023). *J. Synchrotron Rad.* **30**, 746–757. 10.1107/S1600577523003399 PMC10325009 37145139

[bb40] Xingxing, T., Haigang, L. & Guo, Z. (2011). *Acta Opt. Sin.* **31**, 0418001.

[bb41] Xu, H., Yu, X. & Tai, R. (2011). *AIP Conf. Proc.* **1365**, 52–56.

[bb43] Xu, H., Zhou, J., Gong, P. *et al.* (2012). *He Jishu Nucl. Tech.* **35**, 587–590.

[bb44] Yang, Y., Leng, Y. B., Yan, Y. & Chen, Z. (2015). *Chin. Phys. C.* **39**, 097003.

[bb45] Ye, X. X., Ai, H., Guo, Z., Huang, H., Jiang, L., Wang, J., Li, Z. & Zhou, X. (2016). *Corros. Sci.* **106**, 249–259.

[bb46] Zen, H., Adachi, M., Katoh, M., Taira, Y., Yamazaki, J., Hayashi, K., Kimura, S., Tanikawa, T., Hosaka, M., Yamamoto, N., Takashima, Y. & Takahashi, T. (2010). *Proceedings of the 1st International Particle Accelerator Conference (IPAC2010)*, 23–28 May 2010, Kyoto, Japan, pp. 2573-2575.

[bb47] Zhang, L., Xu, Z. & Zhang, X. (2015). *Nucl. Sci. Tech.* **26**, 1–11.

[bb48] Zhao, Z. T., Xu, H. J. & Ding, H. (2015). *Energy*, **3**, 3–51.

[bb49] Zhou, R., Zhang, X., Xu, Z., *et al.* (2011). *He Jishu Nucl. Tech.* **34**, 321–325.

